# Quantifying the amount of greater brain ischemia protection time with pre-hospital vs. in-hospital neuroprotective agent start

**DOI:** 10.3389/fneur.2022.990339

**Published:** 2022-09-13

**Authors:** Vartan Matossian, Sidney Starkman, Nerses Sanossian, Samuel Stratton, Marc Eckstein, Robin Conwit, David S. Liebeskind, Latisha Sharma, May-Kim Tenser, Jeffrey L. Saver

**Affiliations:** ^1^MSTAR Program, Department of Geriatrics, University of California, Los Angeles, Los Angeles, CA, United States; ^2^Stroke Center and Department of Emergency Medicine, University of California, Los Angeles, Los Angeles, CA, United States; ^3^Department of Neurology, University of Southern California, Los Angeles, CA, United States; ^4^Department of Emergency Medicine, University Harbor-UCLA Medical Center, Los Angeles, CA, United States; ^5^Department of Emergency Medicine, University of Southern California, Los Angeles, CA, United States; ^6^Division of Extramural Research, National Institutes of Health/National Institute of Neurological Disorders and Stroke, Bethesda, MD, United States; ^7^Stroke Center and Department of Neurology, University of California, Los Angeles, Los Angeles, CA, United States

**Keywords:** neuroprotection, emergency medical services (EMS), endovascular thrombectomy (EVT), ischemic stroke, clinical trial

## Abstract

The objective of this study is to quantify the increase in brain-under-protection time that may be achieved with pre-hospital compared with the post-arrival start of neuroprotective therapy among patients undergoing endovascular thrombectomy. In order to do this, a comparative analysis was performed of two randomized trials of neuroprotective agents: (1) pre-hospital strategy: Field administration of stroke therapy-magnesium (FAST–MAG) Trial; (2) in-hospital strategy: Efficacy and safety of nerinetide for the treatment of acute ischemic stroke (ESCAPE-NA1) Trial. In the FAST-MAG trial, among 1,041 acute ischemic stroke patients, 44 were treated with endovascular reperfusion therapy (ERT), including 32 treated with both intravenous thrombolysis and ERT and 12 treated with ERT alone. In the ESCAPE-NA1 trial, among 1,105 acute ischemic stroke patients, 659 were treated with both intravenous thrombolysis and ERT, and 446 were treated with ERT alone. The start of the neuroprotective agent was sooner after onset with pre-hospital vs. in-hospital start: 45 m (IQR 38–56) vs. 122 m. The neuroprotective agent in FAST–MAG was started 8 min prior to ED arrival compared with 64 min after arrival in ESCAPE–NA1. Projecting modern endovascular workflows to FAST–MAG, the total time of “brain under protection” (neuroprotective agent start to reperfusion) was greater with pre-hospital than in-hospital start: 94 m (IQR 90–98) vs. 22 m. Initiating a neuroprotective agent in the pre-hospital setting enables a faster treatment start, yielding 72 min additional brain protection time for patients with acute ischemic stroke. These findings provide support for the increased performance of ambulance-based, pre-hospital treatment trials in the development of neuroprotective stroke therapies.

## Introduction

The longer it takes to treat an ischemic stroke, the worse is the outcome (1). The most effective therapy for acute ischemic stroke is restoring cerebral perfusion by endovascular thrombectomy (EVT). However, EVT treatment can only attain reperfusion after a substantial time delay from stroke onset, after the patient or a witness has activated the emergency medical services system, ambulances have responded and identified a stroke in progress, pre-hospital personnel transport the patient to a receiving stroke center, initial brain and vessel imaging are performed, the patient is transported to the neuro-interventional suite, the arterial puncture is performed, catheter systems are navigated to the occluded artery, and an EVT retriever device deployed. As a result, by the time reperfusion is achieved with EVT, the preponderance of patients has already suffered some degree of irreversible infarction, limiting their eventual functional outcome.

Neuroprotective agents have been identified as a class of therapeutics with great promise as a complementary treatment with EVT, slowing infarct progression in the pre-reperfusion period and yielding more salvageable tissue at the time of reperfusion. Treatment by the neuroprotective agent involves the use of drugs or devices with the capacity to interrupt the cellular, biochemical, and metabolic processes that lead to brain injury during ischemia (2).

Two strategies for the delivery of neuroprotective agents prior to EVT have been explored in large randomized clinical trials: (1) pre-hospital initiation by paramedics in the field soon after EMS activation; and (2) in-hospital initiation after imaging has confirmed acute cerebral ischemia. The advantage of pre-hospital initiation is an earlier treatment start, resulting in a longer period in which the brain is under protection prior to EVT. The advantage of an in-hospital start is a more certain diagnosis of acute cerebral ischemia. To inform the decision regarding which approach to take, it is important to know the extent of the time lost—information gained tradeoff between the two strategies. However, to our knowledge, the exact amount of brain protection time gained with the use of pre-hospital vs. in-hospital treatment start has not previously been characterized.

The present study was therefore undertaken with the objective of quantifying the additional amount of time the brain is under protection by a potential neuroprotective agent with pre-hospital compared to in-hospital start by comparing workflow metrics in two exemplar large clinical trials, one of pre-hospital and the other of post-arrival neuroprotection start.

## Materials and methods

This study is a comparative analysis of two randomized clinical trials involving the use of neuroprotective agents. The first trial, that employed the pre-hospital initiation strategy, was the field administration of stroke therapy-magnesium (FAST–MAG), a phase 3, National Institute of Neurological Disorders and Stroke–sponsored, placebo-controlled randomized clinical trial of field-initiated magnesium sulfate in patients with hyper-acute stroke within 2 h after the last known well-time (LKWT) conducted from 2005 to 2013 (3). Participating sites included 40 emergency medical system agencies, 315 ambulances, and 60 acute care receiving hospitals in Los Angeles and Orange counties in California (3). The study protocol was approved by the institutional review board at each pre-hospital and hospital study site (3). Enrollment occurred using explicit informed consent obtained via cellphone conversation between patients on the scene or their legally authorized representatives and enrolling physician-investigators off the scene or under exception from informed consent regulations (3).

The second trial, that employed the in-hospital post-initial imaging strategy, was the efficacy and safety of nerinetide for the treatment of acute ischemic stroke (ESCAPE-NA1) clinical trial. This study took place at 48 acute stroke hospitals in various regions, namely Europe, Asia, and North America between 2017 and 2019 (4). The ESCAPE-NA1 trial was also randomized, double-blind, and placebo-controlled to investigate the efficacy and safety of intravenous nerinetide, specifically in patients with ischemic stroke that were planned to undergo endovascular thrombectomy (EVT) (4). The study was approved by the ethics board at each location and by the responsible regulatory authorities. Informed consent was taken in written form from patients or legally authorized representatives, or via deferred consent under FDA Exception from Informed Consent in Emergency Circumstances regulations (4).

Entry criteria for the current analysis, applied to patients from both trials, were: (1) patient with a final diagnosis of acute ischemic stroke; (2) patient received study agent (either active or placebo) in the field; and (3) patient received endovascular reperfusion therapy after hospital arrival.

To compare drug start and workflow times in the two clinical trials directly, unique time variables were created for analysis. Using published data sets from ESCAPE—the focal ischemia-reperfusion model study in humans and FAST–MAG studies, variables were created for “door to drug,” “drug to puncture,” “door to reperfusion,” “onset to puncture,” and “onset to door” times. All values were quantified using median time values from both studies. “Door to drug” was calculated using the formula “door to puncture” minus “CT to puncture” plus “CT to drug.” “Drug to puncture” time was calculated using the formula “CT to reperfusion” minus “CT to puncture” plus “drug to reperfusion.” “Door to reperfusion” was calculated using the formula “door to puncture” minus “CT to puncture” plus “CT to reperfusion.” “Onset to puncture” was calculated using the formula “onset to drug” plus the difference between “CT to puncture” and “CT to drug.” “Onset to door” was calculated using the formula “onset to puncture” minus “door to puncture”.

### Statistics

The FAST–MAG Trial was performed in an earlier era of endovascular reperfusion therapy than ESCAPE NA-1. At that time, workflow processes to achieve rapid door to puncture were less developed than at present. To avoid having the analysis of the effect of pre-hospital vs. in-hospital neuroprotective agent start upon brain ischemia protection time confounded by unrelated, era-dependent differences in endovascular reperfusion workflow, the lead analysis imputed the observed endovascular workflow times of the ESCAPE-NA1 trial to FAST–MAG patients. Therefore, the “NP start to puncture (modern era)” interval for FAST–MAG patients was derived by combining the actual FAST–MAG “NP start to door” time with the ESCAPE NA-1 “door to puncture” time. In a similar manner, the “door to reperfusion” time interval for patients with FAST–MAG was derived by combining the actual FAST–MAG “NP start to door” time with the ESCAPE NA-1 “door to reperfusion” time.

Patient features in the two trials were delineated with descriptive statistics, using mean and standard deviation for normally distributed variables and median and interquartile range for non-normally distributed variables. Statistical analysis of associations used χ2 tests for binary variables and *t-*tests for linear variables. Two-sided *P* ≤ 0.05 values were considered statistically significant. Because all analyses were considered exploratory, no adjustment for multiplicity was made.

## Results

In the FAST–MAG trial, among 1,041 acute ischemic stroke patients, 44 were treated with endovascular reperfusion therapy (ERT), including 32 treated with both intravenous thrombolysis and ERT and 12 treated with ERT alone. In the ESCAPE-NA1 trial, among 1,105 patients with acute ischemic stroke enrolled with planned ERT, 659 were treated with both intravenous thrombolysis and ERT, and 446 were treated with ERT alone.

The demographic and clinical characteristics of the pre-hospital NP patients treated with EVT (from FAST–MAG) and post-arrival NP patients treated with EVT (from ESCAPE NA-1) patients are shown in Table 1. Both groups were similar in age, in multiple vascular risk factors, and in stroke deficit severity after hospital arrival on the NIH stroke scale. Compared with post-arrival NP patients, pre-hospital NP patients were numerically more frequently female (61.4 vs. 49.7%), more often had a history of atrial fibrillation (45.5 vs. 35.0%), less often were current or recent tobacco users (6.8 vs. 48.9%), and had higher systolic blood pressure on hospital arrival (159 vs. 146 mmHg).

**Table 1:** Demographic and baseline characteristics of patients treated with EVT in FAST–MAG and ESCAPE-NA1.

**Feature**	**FAST-MAG**	**ESCAPE-NA1**
	**(*n* = 44)**	**(*n* = 1,105)**
Age, mean (SD), y	69.3 (12.01)	71
Female, No. (%)	27 (61.4)	549 (49.7)
**Race, No. (%)**		
White	38 (86.4)	889 (80.5)
Black/African American	4 (9.1)	N/A
Asian	2 (4.5)	107 (9.7)
Other	0 (0.0)	109 (9.9)
Hispanic ethnicity, No. (%)	10 (22.7)	N/A
**Medical history, No. (%)**		
Hypertension	33 (75.0)	774 (70.0)
Diabetes	10 (22.7)	218 (19.7)
Hyperlipidemia	20 (45.5)	514 (46.5)
Atrial fibrillation	20 (45.5)	387 (35.0)
Heart disease–ischemic	9 (20.5)	252 (22.8)
Prior Stroke/TIA	5 (11.4)	157 (14.2)
Tobacco use	3 (6.8)	540 (48.9)
Any alcohol use	16 (36.4)	N/A
**SBP, mean (SD), mm Hg**		
Pre-hospital	155.6 (26.4)	N/A
Hospital arrival	158.8 (32.4)	146
**Severity scores**		
Pre-hospital LAMS		
Median (IQR)	5.0 (5.0–5.0)	N/A
Mean (SD)	4.7 (0.8)	N/A
**Hospital arrival NIHSS**		
Median (IQR)	18.5 (11.8–23.3)	17 (12.5–21)

**Figure 1:**
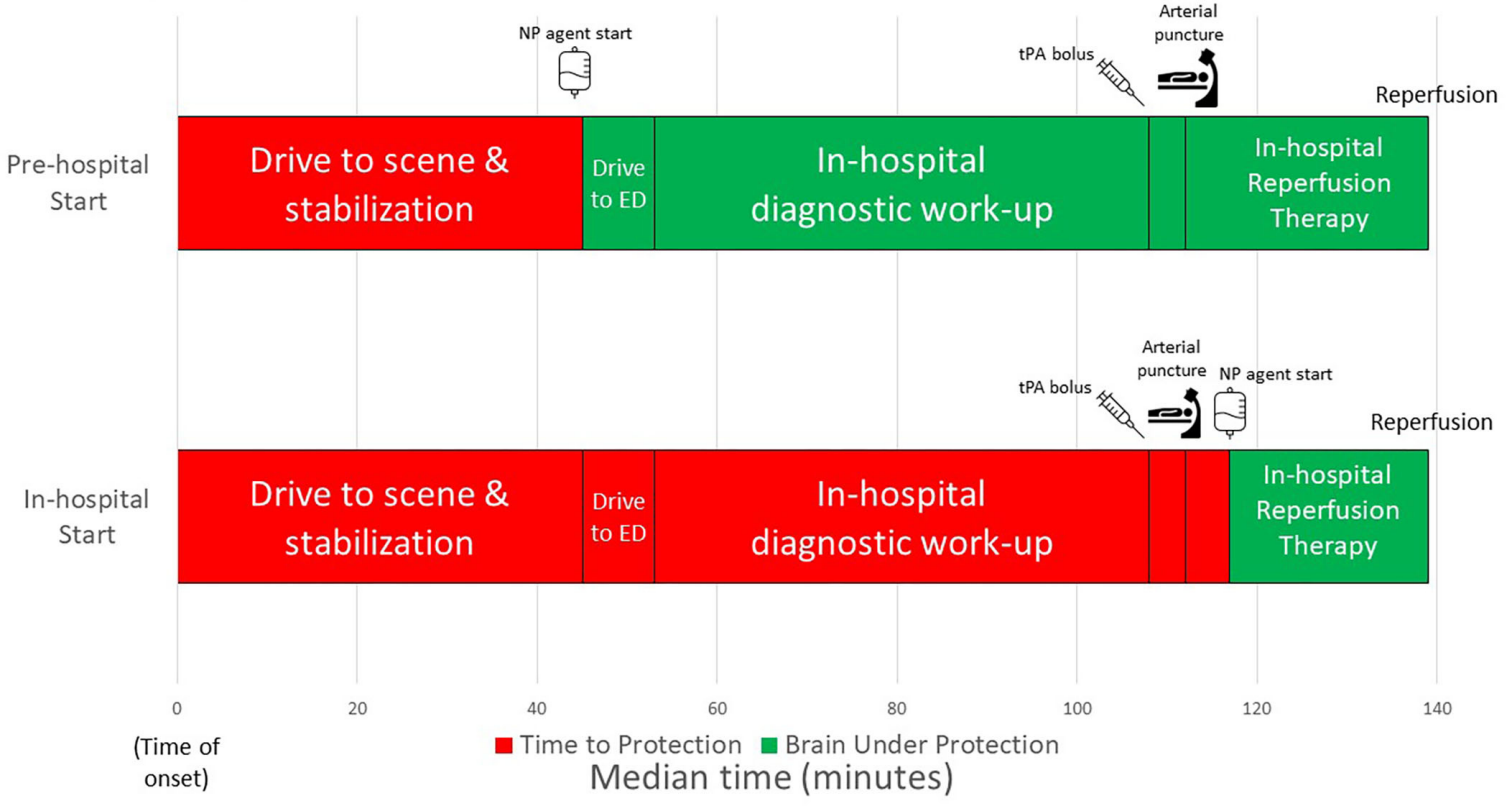
Workflow times comparing neuroprotective start strategies of in-hospital from ESCAPE-NA1 and pre-hospital from FAST-MAG.

The workflow time interval comparisons for the two trials are shown in Table 2; Figure 1. Considering actual start times, the NP agent was initiated 153 min sooner after onset with pre-hospital NP start compared with in-hospital NP trial (agent start 48 min after last known well in FAST–MAG vs. 201 min after last known well in ESCAPE NA-1). The door-to-NP agent needle time in the in-hospital trial was a median of 64 min while in the pre-hospital trial it was median of negative 8 min, as an agent was started prior to emergency department (ED) arrival.

**Table 2:** Time interval results with pre-hospital vs. in-hospital neuroprotective start.

	**Pre-hospital (FAST-MAG)**	**In-hospital (ESCAPE NA-1)**
Last known well to NP agent start	45 min (IQR 38–56)	122 min
ED door to NP agent start	−8 min (IQR −4 to −12)	64 min
NP agent start to puncture	67 mins (IQR 63–71)	−5 min
NP agent start to reperfusion (Time “Brain Under Protection”)	94 min (IQR 90–98)	22 min

Values for the In-hospital column are based on actual ESCAPE-NA1 door-to-NP times and actual ESCAPE NA1 door-to-EVT times. Values for the Pre-hospital column are based on actual FAST–MAG NP-to-door times and the more contemporary ESCAPE NA-1 door-to-EVT times.

As expected, the actual EVT workflow from ED arrival to EVT procedure events was substantially longer in the earlier era FAST-MAG trial compared with the modern era ESCAPE NA-1 trial. Door to puncture was median 215 min in FAST-MAG vs. 59 min in ESCAPE NA-1. The door-to-reperfusion interval in ESCAPE NA-1 was median 86 min while door-to-reperfusion was not recorded in the FAST-MAG trial.

Projecting post-arrival speeds of EVT care in ESCAPE NA-1 to patients treated with NP agent pre-hospital in FAST-MAG, the time from NP agent start to puncture was 72 min sooner with pre-hospital treatment start. The time from NP agent start to reperfusion was 94 (IQR 90–98) min with pre-hospital NP agent start compared with 22 min with in-hospital NP agent start. Therefore, with pre-hospital agent start the total time between NP agent start and expected reperfusion was 72 min greater.

## Discussion

In the present study comparing two neuroprotective treatment trials, delivering a potential neuroprotective treatment pre-hospital compared with post-ED arrival yielded a marked increase in the amount of time between neuroprotective agent start and reperfusion. After correction for disparate workflow speeds in different eras of EVT therapy, pre-hospital agents start conferred 1 h and 12 min more time of “brain under protection”—the interval from drug start to the resolution of ischemic stress. The greater time for the neuroprotective agent to act accrued from the NP agent having an opportunity to work in several additional segments of care, including during the intervals from pre-hospital care to ED arrival, from ED arrival to initial brain imaging, and from brain imaging to puncture.

To highlight the importance of this early treatment method, it is important to understand the biological impact of such an observed time delay. In a typical acute ischemic stroke, 1.9 million neurons, 14 billion synapses, and 7.5 miles of myelinated fibers are lost every minute (1). Considering the longer time that the brain goes without treatment with in-hospital treatment start, pre-hospital start 72 min earlier of a neuroprotective agent that immediately completely protected the ischemic penumbra would save 136 million neurons. In terms of volume, as non-lacunar ischemic strokes typically destroy 5.4 millimeters of tissue per hour, the earlier agent start would save 6.5 ml of brain tissue, a quite substantial volume. At the individual patient level, the faster agent start would result in reduced final disability in 19 of every 100 treated patients (5). In terms of health-related quality of life, the earlier agent start would provide treated patients with an average of 1.2 additional years of healthy life (6).

The current study compared the two most commonly used strategies of NP agent delivery in clinical trials: pre-hospital start vs. in-hospital start after completion of neuroimaging. There are two other strategies of NP agent start in theory, but both are difficult to implement in practice. One is to initiate the NP agent at initial receiving hospitals that are non-thrombectomy centers so that it can act during the time needed for interfacility transfer to a thrombectomy-capable center. The challenge with this strategy is that fewer patients are now arriving at non-thrombectomy centers, due to the increased dissemination of thrombectomy-capable centers and the implementation by regional ambulance systems of direct routing to thrombectomy-capable centers of more severely injured patients (7). The remaining non-thrombectomy-capable centers are not only fewer than in the past, but also often do not have the research infrastructure to implement the initial trial enrollment and agent start. The other potential strategy, applicable to patients who arrived from the field directly to thrombectomy-capable centers, is to start the NP immediately upon ED arrival and prior to brain imaging. But practical obstacles to this strategy are the difficulties in eliciting research informed consent, performing randomization, and preparing and delivering the study drug in the first minutes after arrival as the stroke team is at that time intensely focused on the speedy performance of conventional acute care: brain imaging, delivery of intravenous thrombolytics, and the start of endovascular thrombectomy. Therefore, the current study compares the two strategies for research agent NP initiation that predominate in current trials of cerebroprotection.

It is important to emphasize that the current study results apply only to clinical trials of neuroprotective agents that are safe for use in both ischemic and hemorrhagic stroke. Neuroprotective agents safe in ischemic stroke but harmful in hemorrhagic stroke cannot be tested in the pre-hospital setting, as the study agent could only safely be started after brain imaging. Agents currently under development that are suitable for pre-hospital, post-arrival, or during procedures have been recently reviewed (8).

The time to treatment data in the in-hospital trial analyzed in the current investigation was consonant with in-hospital neuroprotective trials conducted in the endovascular thrombectomy era. In a randomized trial of uric acid performed in the endovascular era, the neuroprotective agent was started 35 min after the start of intravenous alteplase, indicating a similar start time as in ESCAPE-NA1 (9). In a randomized trial of p3K3A-APC, treatment initiation was permitted up to 120 min after the arterial puncture, a longer time period than in ESCAPE-NA1 (10).

The present study has limitations. First, as the FAST-MAG trial was performed at a time when only early EVT technology was available and workflow was slow for EVT patients, the lead analysis was based upon ESCAPE-NA1 EVT workflow speeds projected upon FAST-MAG patients, rather than the actual workflow pace in FAST–MAG. Second, a drawback of pre-hospital standard ambulance neuroprotective trials is that they enroll a heterogeneous group of patients, including patients with non-LVO ischemic stroke and with intracerebral hemorrhage. This aspect is not reflected in the current analysis focused on EVT-receiving patients. However, the justification for testing neuroprotective agents in pre-hospital trials is that the longer time for agents to exert treatment effects offsets the greater heterogeneity of patients enrolled. The current study provides useful quantitative data for evaluating this tradeoff.

In conclusion, this study demonstrates that starting a neuroprotective agent in the pre-hospital setting, compared with after hospital arrival, greatly increases the period of time that the brain is “under protection” prior to reperfusion. These results support the use of the ambulance-based pre-hospital start of neuroprotective agents in clinical trials of agents that are safe for use in both ischemic and hemorrhagic stroke.

## Data availability statement

The data analyzed in this study was obtained from National Institute of Neurological Disorders and Stroke (NINDS; https://www.ninds.nih.gov/current-research/research-funded-ninds/clinical-research/archived-clinical-research-datasets), the following licenses/restrictions apply: To request a dataset, the NINDS Data Request Form must be completed and sent to the NINDS Clinical Research Liaison. Requests to access these datasets should be directed to the NINDS Clinical Research Liaison, CRLiaison@ninds.nih.gov.

## Ethics statement

The studies involving human participants were reviewed and approved by University of California, Los Angeles. The patients/participants provided their written informed consent to participate in this study.

## Author contributions

VM and JS contributed to the conception, design of the study, and performed the statistical analysis. SSta, NS, ME, DL, LS, M-KT, and JS performed data collection. SStr and RC provided administrative and scientific support for the study. VM wrote the first draft of the manuscript. All authors contributed to manuscript revision, read, and approved the submitted version.

## Funding

This research was funded by the Medical Student Training in Aging Research Program, the National Institute on Aging (T35AG026736), and the National Institute of Neurologic Diseases and Stroke (U01 NS44364).

## Conflict of interest

The authors declare that the research was conducted in the absence of any commercial or financial relationships that could be construed as a potential conflict of interest.

## Publisher's note

All claims expressed in this article are solely those of the authors and do not necessarily represent those of their affiliated organizations, or those of the publisher, the editors and the reviewers. Any product that may be evaluated in this article, or claim that may be made by its manufacturer, is not guaranteed or endorsed by the publisher.
